# Efficacy of *Phellinus linteus* (sanghuang) extract for improving immune functions: Study protocol for a randomized, double-blinded, placebo-controlled pilot trial

**DOI:** 10.1097/MD.0000000000018829

**Published:** 2020-01-17

**Authors:** Ga Hyeon Jung, Jae Hui Kang

**Affiliations:** a Department of Acupuncture & Moxibustion Medicine, College of Korean Medicine, Daejeon University; b Department of Acupuncture and Moxibustion Medicine, Cheonan Korean Medicine Hospital of Daejeon University, Chungcheongnamdo, Republic of Korea.

**Keywords:** immune function, *Phellitus linteus*, randomized controlled trial, study protocol

## Abstract

**Background:**

With the increase in the prevalence of chronic diseases, enhancing immunity has become an important challenge. Health functional foods, such as *Phellitus linteus* (PL), are known to improve immune function. In previous laboratory studies, it has been reported that PL has anticancer activities. In this study, we will evaluate if PL improves immune functions in adults with reduced immunity and identify its effects on immunity factors.

**Methods:**

This study will be a single-center, randomized, double-blinded, and placebo-controlled trial. Study participants will be randomly allocated to 3 experimental groups: those taking PL 1000 mg, PL 2000 mg, or placebo. Total duration of the clinical trial will be 8 to 10 weeks. Study participants will have follow-up visits every 4 weeks and the effect and safety will be assessed at the second and third visits. All participants will be asked to maintain a dosage schedule for this protocol. The primary outcome will be natural killer (NK) cell activity and the secondary outcomes will include peripheral white blood cell (WBC) count; levels of tumor necrosis factor (TNF)-α, interferon (IFN)-γ, and interleukin (IL)-1β, IL-2, IL-6, and IL-12 to assess autoimmune reactivity; and levels of immunoglobulin (Ig)G1, IgG2, and Immunoglobulin M (IgM) to assess allergic activity. Repeated measure analysis will be used to measure primary efficacy based on full analysis set.

**Discussion:**

This study has limited inclusion and exclusion criteria and a well-controlled intervention. It will be the first randomized controlled trial to assess the efficacy and safety of PL in adults with reduced immunity. This study will provide insights into the mechanisms underlying the immune-stimulating effects of PL and lay the groundwork for further studies.

## 1 Introduction

As the prevalence of chronic diseases is increasing, treatments for enhancing immunity are receiving more attention.^[1]^ Immune-related treatments can involve 2 approaches: enhancing an individual's immunity, which aims to strengthen the body's biological defense mechanisms and alleviating hypersensitivity, which aims to control allergic reactions. It is known that along with aging, immunological biomarkers deteriorates, it can increase the frequency and severity of inflammation and the incidence of self-immune diseases and cancer.^[2]^

As nutrition plays an important role in maintaining the immune functions of the human body, increasing attention has been paid to health functional foods, as they are known to have effects on improving immune function.^[3]^ One health functional food, *Phellitus linteus* (PL), belongs to the fungal family *Hymenochaetaceae* and grows on mulberry trees.^[4]^

In previous studies, the anticancer activity of PL has been examined. In 1968, Ikekawa et al^[5]^ reported that hot water extracts from *Polyporaceae* mushrooms inhibit the growth of sarcoma-180, and among those, PL extract was highly effective. Oh^[6]^ reported that proteoglycan separated from PL increases the production of interleukin (IL)-12 (IL-12) and interferon-γ (IFN-γ). Kong et al^[7]^ also reported that water extract of PL may be associated with augmentation of natural killer (NK) cell function in cancerated hosts. PL is known to suppress tumor growth, invasion, and angiogenesis^[8]^ and PL is also known to cause tumor regression by inducing apoptosis.^[9]^ Several other studies have reported that PL stimulates humoral and cell-mediated immunity by stimulating T-lymphocytes, NK cells, macrophages, and B-lymphocytes.^[10–12]^

This study protocol was designed to verify the safety and efficacy of PL on the immune system using a single-center, randomized, placebo-controlled, and double-blinded clinical pilot trial.

## 2 Methods

### 2.1 Study design

This study has been designed as a double-blinded, single-center, randomized clinical trial for investigating the efficacy of PL in immune functions. A total of 30 subjects with reduced immunity will be recruited from outpatients at DUCKMH through advertisements posted on bulletin boards at hospitals and apartments. Recruitment commenced in October 2019 and the trial is expected to end in December 2020. All participants will receive a full written explanation of the study's protocol and an informed consent form.

During screening, medical history within the past 6 months and drugs taken within the past 4 weeks will be recorded. Additionally, blood tests for liver function, complete blood count, and immune function will be performed.

A total of 30 adults with reduced immunity who meet the eligibility criteria for this study will be randomly assigned in a 1:1:1 ratio to treatment group 1 (PL 1000 mg), treatment group 2 (PL 2000 mg), or control group (placebo). The intervention will begin within 2 weeks of the screening visit.

**Figure 1:**
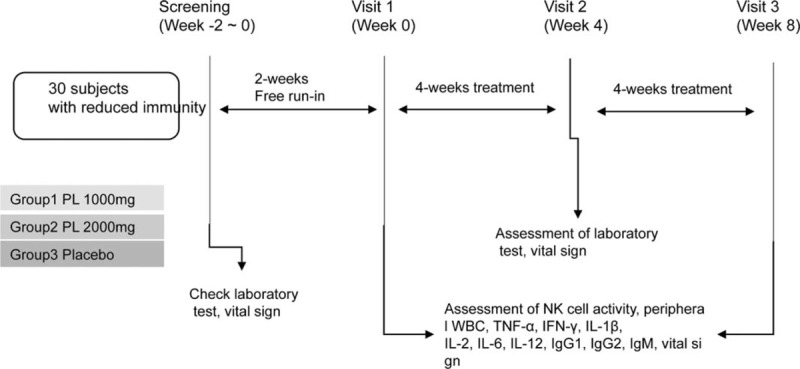
Study design for PL clinical trial. The treatment or control groups will be administered PL 1000 mg or PL 2000 mg or placebo. The subjects will receive PL 1000 mg or PL 2000 mg or placebo for 8 weeks and will visit every 4 weeks. PL = *Phellitus linteus*.

Based on their study group, the subjects will be administered PL 1000 mg, PL 2000 mg, or placebo for 8 weeks. The total duration of the clinical trial will be 8 to 10 weeks. Subjects will have follow-up visits every 4 weeks, and the efficacy and the safety will be assessed at the second and third visits. The study flow chart is presented in Fig. 1.

### 2.2 Inclusion and exclusion criteria

The inclusion criteria are as follows: age 20 to 65 years; peripheral white blood cell (WBC) count between 3 × 10^3^/μL and 8 × 10^3^/μL; history of upper respiratory infection (URI) symptoms twice or more within 1 year prior to the study; and written consent for participating in the trial.

The exclusion criteria are as follows: clinically significant cardiovascular, immune, respiratory, hepatobiliary, renal and urological, nervous, musculoskeletal, psychological, infectious, and/or neoplastic diseases that are currently being treated; uncontrolled hypertension or diabetes mellitus; history of vaccination within 1 month of the study; aspartate aminotransferase (AST) or alanine aminotransferase (ALT) levels over 120 IU/L; creatinine over 2.4 mg/dL (man) or 1.8 mg/dL (woman); intake of health functional foods that may affect the immune system within 2 weeks of the study; severe gastrointestinal symptoms like heartburn and indigestion; pregnancy or breastfeeding; food allergies to foods related to PL; participation in the other clinical trials during the study; intake of drugs of other clinical trial within 4 weeks of the study.

### 2.3 Sample size calculation

We have demonstrated in both in vitro and in vivo experiments that PL has immunological effects. To determine the minimum effective concentration, PL was administered at various concentrations, and 200 and 400 mg/kg of PL was administered to mice for 7 days. Based on the United States Food and Drug Administration (FDA)'s guidelines for calculating the effective dose in humans, we used body surface area to translate the effective dose in animal studies to human studies. Based on these calculations, the effective dose in the human body was set at 960 and 1920 mg. For ease of production and intake, the dose was set at 1000 and 2000 mg. According to Whitehead et al^[13]^ in preliminary parallel-designed studies when effect size is considered medium, the empirically optimal sample size is 10 persons per group, due to considerations of feasibility, estimated average, and dispersion accuracy. Therefore, we designed a pilot study with 10 subjects in each group.

### 2.4 Randomization and blinding procedures

Randomization will be performed by stratified block randomization method in treatment groups 1 and 2 and control group at a ratio of 1:1:1 to ensure a balanced distribution of the 3 groups. Subjects who meet all registration criteria will be randomly assigned to a randomization identification code (e.g., PLI-R-001, PLI-R-002, …, PLI-R-030) in the order generated by a computer randomization program. The randomization number will be given to each participant, and it will indicate whether PL 1000, 2000, or placebo is to be given. As this pilot study is designed to be double-blinded, the participants, researchers, and assessors collecting the data will be blinded to the group allocation. Information about intervention assignment will be stored in the third statistical department. The randomization code will be placed in an opaque envelope and stored at the hospital. With the exception of disclosure to individual patients with serious adverse events (SAE), randomization and blinding will not be disclosed to researchers until the end of the trial.

### 2.5 Intervention

All eligible participants will receive treatment according to their allocated group, either PL 1000 mg, PL 2000 mg, or placebo, during the 8-week treatment period. To maintain the double-blinded nature of the study, PL 1000 mg, PL 2000 mg, and placebo will be manufactured to be similar in appearance by Hankookshinyak Pharmaceutical Co. (Nonsan, Republic of Korea) and 2 tablets will be taken orally twice a day. The subjects will be evaluated twice in 8 weeks (at 4 and 8 weeks).

### 2.6 Outcome measures

The primary outcome is NK cell activity, which will be used to assess immune reactivity. The secondary outcomes are peripheral WBC count; levels of TNF-α, IFN-γ, IL-1β, IL-2, IL-6, and IL-12 to assess autoimmune reactivity; and levels of IgG1, IgG2, and IgM to assess allergic activity. URI symptoms will also be recorded. All outcome measures will be observed at every visit. For the safety of the participants, we will conduct laboratory tests, such as liver function panels, complete blood counts, and electrolyte levels, and we will evaluate vital signs. Safety assessments will be conducted at the second and third visits. Participants’ data will be anonymized and coded by a specific program.

### 2.7 Data collection and monitoring

During the screening period, the subjects will fill out a questionnaire about their sociodemographic characteristics, provide a medical history from the last 6 months, provide a drug history from the past 4 weeks, and undergo laboratory tests. Personal information and data collected during the screening period will be managed by the hospital. Monitoring of data and research performance will be performed regularly by Jeneolurl Bio Taek Co., Ltd. (Pusan, South Korea). The final exam dataset will be accessible to statisticians and key investigators. The results of this study will be published in a peer-reviewed article.

### 2.8 Statistical analysis

Statistical analysis will be performed using the principle of full analysis set. For intention-to-treat (ITT) analysis, missing values will be analyzed by last observation carried forward (LOCF). The baseline values before treatment and the changes in NK cell activity at 4 weeks and after treatment will be measured for the 2 experimental groups and the control group. If the data follow a normal distribution, an independent *t* test will be performed to test the differences between 2 groups of continuous variables. If not, Mann–Whitney *U* test will be performed. Paired *t* tests will be performed to test the differences between groups if the data follow a normal distribution. If not, Wilcoxon signed-rank test will be performed. The test of normality for continuous variables will be analyzed using the Shapiro-Wilk test. The confidence level will be set at 5%. All statistical analyses will be performed using SPSS Statistics for Windows Version 20.0 (IBM Corp., Armonk, NY).

Drinking alcohol, which can affect the weekly evaluation, is considered a management variable, and if there is a difference between groups, an analysis of covariance (ANCOVA) will be performed. Descriptive statistics, such as number of subjects, mean, standard deviation, minimum, median, and maximum will be presented for baseline, at each visit, and at final evaluation. Change from baseline to final evaluation will be reported for continuous data, clinical laboratory tests, and vital signs. For categorical data, a division table will be provided. If necessary, 95% confidence intervals will be calculated. No intermediate analysis will be performed.

Demographic variables measured during the screening period will be summarized for each treatment group. Assays for comparison of drug administration groups will be performed. For this purpose, the continuous data will be tested using the *t* test or Wilcoxon rank-sum test. Categorical data will be analyzed using a chi-square test or Fisher exact test.

The safety assessment will calculate the rate of adverse events. Continuous data, such as comparisons between groups, individual laboratory test results, and biomarkers will be analyzed using paired *t* tests or *t* tests with 95% confidence intervals within the group to see if there are any changes compared with the baseline. If normality is not met, nonparametric statistics (Wilcoxon signed-rank test or Wilcoxon rank-sum test) will be performed. Categorical data will suggest frequencies and ratios for each category. The difference between groups will be analyzed using chi-square test and Fisher exact test.

### 2.9 Withdrawal and dropout

If subjects do not meet the inclusion or exclusion criteria or if they withdraw their consent, they will be excluded from the study. The researcher will report the reason for the interruption and if each participant completed the study.

### 2.10 Safety

Occurrence of side effects will be assessed at each visit. Subjects will be monitored for undesirable or unintended symptoms, signs, and diseases. The number and ratio of subjects who have experienced adverse event will be counted and calculated.

### 2.11 Ethics

This study design is based on the Helsinki Declaration and the Korean Clinical Practice Guidelines. This study has been approved by the Korean Institutional Review Board of DUCKMH (number DJUMC-2019-BM-10-1). This study protocol is registered with the Korean National Clinical Research Information Service (CRIS-KCT0004272). In case of serious adverse events, subjects will be required to withdraw. They will be reported to the Institutional Review Board of the hospital. Any participant in this study may withdraw consent or voluntarily cease to participate at any time for any reason.

## 3 Discussion

The immune system is an organization of cells and molecules with specialized roles in defending against infection. Innate responses occur regardless of how many times the infectious agent is encountered, whereas adaptive responses continually improve with repeated exposure to a given infection. The innate responses use phagocytic cells (i.e., neutrophils, monocytes, and macrophages) that release inflammatory mediators (i.e., basophils, mast cells, and eosinophils), and NK cells.^[14]^

NK cells are effector lymphocytes of innate immunity endowed with constitutive cytolytic functions. Moreover, NK cells also show efficacy against assaults like viral infections and tumor development.^[15]^ It is important to include assays of NK cell markers, peripheral WBCs, TNF-α, IFN-γ, IL-1β, IL-2, IL-6, IL-12 as immunological biomarkers, as well as IgG1, IgG2, and IgM as allergic biomarkers.

In previous studies, PL has been shown to have anticancer effects through stimulating immune function. This study is the first to be conducted that will determine the appropriate dosage and duration of PL administration in human subjects. Although our sample size is small, and resultant biases may occur, this study may provide valuable insights into the mechanisms underlying the immune-stimulating effects of PL and will lay the foundation for further studies on the effect of PL on symptoms in patients with immune problems.

## Author contributions

JHK designed, administrated, and supervised the study protocol and critically revised the manuscript. GHJ developed the protocol and drafted the manuscript. Both authors have read and approved the final manuscript.

Ga Hyeon Jung orcid: 0000-0002-7759-0972.

## References

[1] Lee SJ Li SS Kim DH A Study on the trends of Meebyung research. J Korean Med Classics 2010;23:23–34.

[2] DelaRosa O Pawelec G Peralbo E Immunological biomarkers of ageing in man: changes in both innate and adaptive immunity are associated with health and longevity. Biogerontology 2006;7:471–81. 16957868 10.1007/s10522-006-9062-6

[3] Ryu HS Kim J Kim HS Enhancing effect of Sorghum bicolor L. Moench (Sorghum, su-su) extracts on mouse spleen and macrophage cell activation. Korean J Food Nutr 2006;19:176–82.

[4] Park WH Lee HD Illustrated Book of Korean Medicinal Mushrooms. Seoul, Korea: Kyo-Hak Publishing Co.; 1999.

[5] Ikekawa T Nakanishi M Uehara N Antitumor action of some basidiomycetes, especially Phellinus linteus. Gann 1968;59:155–7. 5723060

[6] Oh WK Immuno-Nodulating Effects and Anti-tumor Activities of Proteoglycan Isolated from Phellinus Linteus. Wonkwang University Master's Degree. 2003.

[7] Kong YY Lee KK Nam SY Experimental studies on activity of the cultivated Mycelia of Phellinus linteus. Kor J Pharmacogn 1991;22:233–9.

[8] Song KS Li G Kim JS Protein-bound polysaccharide from Phellinus linteus inhibits tumor growth, invasion, and angiogenesis and alters Wnt/b-catenin in SW480 human colon cancer cells. BMC Cancer 2011;11:307. 21781302 10.1186/1471-2407-11-307 PMC3154178

[9] Tsuji T Du W Nishioka T Phellinus linteus extract sensitizes advanced prostate cancer cells to apoptosis in athymic nude mice. PLos One 2010;5:e9885. 20360989 10.1371/journal.pone.0009885 PMC2847601

[10] Kim HM Han SB Oh GT Stimulation of humoral and cell mediated immunity by polysaccharide from mushroom Phellinus linteus. Int J Immunopharmac 1996;18:295–303. 10.1016/0192-0561(96)00028-8 8933208

[11] Oh GT Han SB Kim HM Immunostimulating activity of Phellinus linteus extracts to B-lymphocyte. Arch Pharm Res 1992;15:379–81.

[12] Lee JH Cho SM Song KS Immunostimulating activity and characterization of polysaccharides from Mycelium of Phellinus linteus. J Microbiol Biotechnol 1996;6:213–8.

[13] Whitehead AL Julious SA Cooper CL Estimating the sample size for a pilot randomised trial to minimise the overall trial sample size for the external pilot and main trial for a continuous outcome variable. Stat Methods Med Res 2016;25:1057–73. 26092476 10.1177/0962280215588241 PMC4876429

[14] Delves PJ Roitt IM The immune system. N Engl J Med 2000;343:37–49. 10882768 10.1056/NEJM200007063430107

[15] Vivier E Raulet DH Moretta A Innate or adaptive immunity? The example of natural killer cells. Science 2011;331:44–9. 21212348 10.1126/science.1198687 PMC3089969

